# Noradrenergic Regulation of Central Amygdala in Aversive Pavlovian-to-Instrumental Transfer

**DOI:** 10.1523/ENEURO.0224-17.2017

**Published:** 2017-10-24

**Authors:** Vincent D. Campese, Jose M. Soroeta, Elena M. Vazey, Gary Aston-Jones, Joseph E. LeDoux, Robert M. Sears

**Affiliations:** 1 Center for Neural Science, New York University, New York, NY 10003; 2 Department of Biological Sciences, University of Arkansas, Fayetteville, AR 72701; 3 Department of Biology, University of Massachusetts, Amherst, MA 01003; 4 Brain Health Institute, Rutgers University, Piscataway, NJ 08854; 5 Emotional Brain Institute, Nathan Kline Institute for Psychiatric Research, Orangeburg, NY 10962; 6 Department of Child and Adolescent Psychiatry, New York University Langone School of Medicine, New York, NY 10016

**Keywords:** central amygdala, expression, locus coeruleus, motivation, norepinephrine, PIT

## Abstract

The neural mechanisms through which a Pavlovian conditioned stimulus (CS) elicits innate defense responses are well understood. But a Pavlovian CS can also invigorate ongoing instrumental responding, as shown by studies of aversive Pavlovian-to-instrumental transfer (PIT). While the neural circuitry of appetitive PIT has been studied extensively, little is known about the brain mechanisms of aversive PIT. We recently showed the central amygdala (CeA) is essential for aversive PIT. In the current studies, using pharmacology and designer receptors in rodents, we demonstrate that noradrenergic (NE) activity negatively regulates PIT via brainstem locus coeruleus (LC) activity and LC projections to CeA. Our results provide evidence for a novel pathway through which response modulation occurs between brainstem neuromodulatory systems and CeA to invigorate adaptive behavior in the face of threat.

## Significance Statement

The results reported herein use Pavlovian-to-instrumental transfer (PIT), a test of the motivational value of a conditioned stimulus (CS), to study noradrenergic (NE) contributions to aversive motivation. During transfer tests, a shock-paired cue elevates separately trained shock-avoidance responding. Designer receptor excitation of locus coeruleus (LC) and its projections to central amygdala (CeA) before transfer testing eliminated the effect of the cue on shock-avoidance behavior. These findings provide the first evidence that noradrenaline negatively regulates this phenomenon in aversive motivation.

## Introduction

Much has been learned about the neural basis of threat processing using Pavlovian threat conditioning (PTC; LeDoux, 2015). In this procedure, a neutral conditioned stimulus (CS) paired with a noxious unconditioned stimulus (US) such as footshock comes to elicit conditioned responses (CRs), such as freezing behavior (Blanchard and Blanchard, 1972; Bolles and Fanselow, 1980). In addition to eliciting simple CRs, Pavlovian cues can also motivate or enhance other actions that are associated with the US through prior experience. For example, in Pavlovian-to-instrumental transfer (PIT) a previously shock-paired CS, rather than generating passive freezing CRs, increases the rate of ongoing active footshock avoidance behavior (e.g., two-way shuttling; Campese et al., 2013; also see Rescorla and Lolordo, 1965; Weisman and Litner, 1969; Overmier and Payne, 1971; Overmier and Brackbill, 1977; Patterson and Overmier, 1981). In recent work, we have begun to explore the neural basis of aversive PIT. Using brain lesions to disrupt neural activity, we found that the facilitative effect of the aversive CS on avoidance is impaired by damage to specific nuclei of the amygdala (Campese et al., 2014, 2015; McCue et al., 2014). Thus, damage to the lateral, central or medial nucleus, but not to the basal nucleus, disrupted aversive PIT.

In appetitive PIT, dopamine release in the striatum modulates instrumental performance (Corbit and Balleine, 2011; Ostlund et al., 2011; Shiflett and Balleine, 2011; Wassum et al., 2011; Laurent et al., 2014). In contrast, although it is well established that noradrenergic (NE) neuromodulation in the amygdala regulates aversive learning (Murchison et al., 2004; Gertner and Thomas, 2006; Bush et al., 2010; Tully and Bolshakov, 2010; Johansen et al., 2011; Ferry and Quirarte, 2012; Sears et al., 2014; Schiff et al., 2016), a role for NE in aversive PIT is not known.

To express aversive PIT, CS-elicited freezing responses must be suppressed and avoidance must be increased. The central nucleus of the amygdala (CeA) has been shown to be involved in both of these processes (Campese et al., 2013; Moscarello and LeDoux, 2013). CeA also receives NE inputs from brainstem locus coeruleus (LC) and expresses NE receptors (Kravets et al., 2015). Therefore, in the present study, we explored the contribution of NE to aversive PIT. We first used systemic pharmacology to show a role for NE in PIT. We then examined the effect of increasing NE release from the LC in the CeA on PIT. Terminal manipulations of NE in the CeA were accomplished using designer receptors exclusively activated by designer drugs (DREADDs). These studies suggest that NE release in CeA plays a critical role in CS-elicited behavior and that this neuromodulatory system negatively regulates aversive PIT.

## Materials and Methods

### Subjects

Forty-eight male Sprague Dawley rats, purchased from Hilltop Lab Animals, were used for experiment 1 and eighteen for experiment 2. Subjects weighed ∼275 g at the start of experimentation. Rats were housed in standard Plexiglas cages on a 12/12 h light/dark cycle. Subjects had free access to food and water while in their home cages, which were lined with paper bedding. Animal care and housing met the current standards of the International Association for Assessment and Accreditation of Laboratory Animal Care. The University Animal Welfare Committee at New York University approved all procedures reported herein.

### Apparatus

Pavlovian conditioning took place in context A, a set of standard training chambers manufactured by Coulbourn Instruments (26 × 28 × 20 cm, length × width × height; model no H10-11R- TC) with stainless steel grid floors to deliver the footshock US and an 8-Ω speaker for the 30-s 5-kHz tone CS presentations. Avoidance training and PIT testing took place in context B, which was a two-way shuttle chamber (50.8 × 25.4 × 30.5 cm, model no H10- 11R-SC) also manufactured by Coulbourn. Stainless steel grid floors similar to those in context A presented the footshock US and had an 8-Ω speaker on each side of the chamber to present the tone CS. All chambers were housed in light and sound attenuating shells. Follow-up tests for CS-elicited freezing were conducted in context C. Context C was another set of standard training chambers also manufactured by Coulbourn in a different room and made different from context A by insertion of striped patterns on the plastic walls and peppermint scent (Dr. Bronner’s Magic Soaps) in the waste pan. Additionally, the floor was made from thin mesh-wiring.

### Procedure

Subjects were trained using the procedure developed in our lab to study aversive PIT (Campese et al., 2013). The studies included three main phases: (1) PTC, (2) unsignaled Sidman active avoidance (USAA) training; and (3) PIT testing. An additional test was included where the CS was presented outside of the avoidance context (context B). For experiment 1, subjects were given intraperitoneal (IP) treatment with propranolol, procaterol or the saline vehicle before PIT tests. These pharmacological treatments were also administered before the test for CS-elicited freezing in context C. For experiment 2, following recovery from surgical treatments for viral injections subjects underwent training and testing for PIT as described above. Before tests subject were treated with vehicle or clozapine-N-oxide (CNO) IP in a counterbalanced fashion. Subjects were then retrained and implanted with guide cannula targeting the CeA and given further retraining and testing. These final transfer tests were preceded by intracranial drug treatments but otherwise identical to the previous tests. Finally, subjects were tested for CS-elicited freezing in a nonavoidance context (context C) following IP drug treatments.

### PTC

Subjects first received PTC in context A where, following a 5-min baseline, the 30-s tone CS coterminated with a 1 s duration footshock US (0.7 mA). There were three trials separated by a 180-s intertrial interval.

### USAA

Starting on day 2 of the study, subjects underwent 15 sessions of USAA in context B. There was one session per day with four or five sessions per week and each session was 25 min in duration. During these sessions, shuttle responses were reinforced by extension of shock free periods. One-second 0.7-mA footshocks were delivered every 5 s unless a shuttle response was performed. Shuttling during the shock terminated the event (i.e., escape), while shuttling between shocks (i.e., avoidance) produced a shock free interval of 30 s. All shuttle responses were accompanied by a 0.3-s blinking house light as a feedback cue (for more information, see Lazaro-Munoz et al., 2010). Due to a computer error during experiment 1, training data from day 11 of USAA training were not recorded. Therefore, block 4 in the analysis only includes two sessions. Video recording issues resulted in the loss of two subjects’ footage for USAA freezing. One of these was from the propranolol group, the other from the procaterol group, though subjects had not yet been treated. Subjects that failed to meet the training criteria were eliminated from the study following day 10 of USAA. For inclusion, a subject was required to reach at least twenty avoidance responses in two consecutive training sessions within the first ten days of this phase (for more information, see Lazaro-Munoz et al., 2010).

### PIT testing I

PIT testing took place over two consecutive days in two sessions in context B following completion of USAA training. These tests used parameters established in prior work that produce strong and reliable PIT effects while still maintaining uniform response requirements during transfer (Campese et al., 2013). In each test, following a 15-min baseline shuttling period the CS was evaluated for its capacity to augment instrumental responding (i.e., shuttling) in a single trial. There were no shocks delivered and shuttle responses still produced the blinking house light feedback cue throughout the test phase, including during the CS trials. The tone was presented to each subject when shuttle response rates fell below two responses per minute for a full 2 min following the mandatory 15-min baseline. The tone then remained on until ten shuttle responses were performed, at which point the CS terminated and the houselights turned off. The following day this was repeated. For experiment 1 PIT testing was conducted as described above except that each of these tests were preceded 15 min earlier by systemic injections of propranolol, procaterol or the saline vehicle. For experiment 2, PIT tests were conducted in the same way except for the pretest drug treatments. Instead of receiving β-receptor drugs 15 min before tests, subjects received IP treatment with CNO (2 mg/kg; Sigma Aldrich) or the saline vehicle only for the first two tests. Additionally, for experiment 2, following one week, an additional two tests were conducted with reversed drug assignments. Subjects were matched on USAA performance for assignment into these conditions.

### Retraining (experiment 2). Pre-/postcannulation retraining

The day following the fourth PIT test for experiment 2, subjects received a session of PTC retraining identical to the session described above for day 1 of the study. Over the next two days, subjects then received an additional two USAA training sessions run identically to those described above. These retraining sessions were done (1) to avoid a floor effect for later testing since pilot data we have collected suggests PIT can extinguish over multiple tests and (2) to provide a performance measure against which to compare USAA following cannula implants. Pilot data we have collected suggest that cannula implants in CeA can interfere with USAA acquisition and PIT. In the current study, we compared a third USAA retraining session conducted following recovery from cannula implants to the presurgical sessions to confirm that posttraining implants did not interfere with USAA behavior. Aside from the timing in relation to surgery, these sessions did not differ.

### Infusions and PIT testing II

Before the tests following the retraining phase, infusions into the CeA were made using a Harvard Apparatus pump (PHD 22/2000). Each hemisphere was infused simultaneously with 0.3 μl of CNO (1 mg/ml) or the saline vehicle at a rate of 0.15 μl/min. Each round of testing included two individual test sessions as described above and the two rounds were separated by one week to encourage response recovery. Infusions were counterbalanced over these tests such that half of the animals received vehicle and the other half CNO for the first round of these postoperative tests, and the assignments were reversed the following week for the second round. Infusion assignments were made orthogonal to IP drug assignments from the first round of tests. Half of the CNO-vehicle-treated subjects over the first round of IP tests received CNO-vehicle treatment during the intracranial infusion tests while the other half received vehicle-CNO treatment. The same was true of animals treated vehicle-CNO during the first round of tests. Otherwise, these tests were as described above.

### CS-elicited freezing test

One week after PIT testing in experiment 1, subjects underwent an additional test session in context C, also preceded by systemic drug treatments using the same assigned groups, where three trials of the CS were presented under extinction conditions and freezing was evaluated in the absence of the shuttle response. These sessions were identical to the PTC sessions, but did not include any footshock. For experiment 2, this was done later, following intracranial testing and used a slightly different approach. Subjects received two identical test sessions in the standard nonavoidance context C preceded 20 min earlier by IP CNO (2 mg/kg) or vehicle treatment after both rounds of PIT testing concluded. These assignments were counterbalanced to produce within-subjects measure of the effects of CNO and further balanced with regard to earlier assignments across PIT testing.

### Surgery

Adeno-associated virus (AAV) injections in LC were achieved using procedures previously described (Sears et al., 2013). The viral vector (AAV9.PRSx8.hM3Dq-mCherry.WPRE.rBG) was subcloned and packaged by the University of Pennsylvania Viral Vector Core in AAV2/9. The PRSx8 promoter was used to restrict expression of the HA-tagged hM3Dq DREADD gene to NE neurons in the LC area (Vazey and Aston-Jones, 2014). Animals were anesthetized using a mixture of ketamine (100 mg/kg, i.p.) and xylazine (10 mg/kg, i.p.), with supplementation as needed, along with buprenorphine–HCl (0.02 mg/kg, s.c.) for analgesia, and placed in a stereotaxic apparatus (David Kopf Instruments). The skull was exposed and the LC targeted using the following coordinates (interaural: −0.72 Anterior-Posterior, ±1.35 Medial-Lateral, −7.5 Dorsal-Ventral from skull). AAV (2 μl/side) was delivered via a Hamilton Neuros syringe (5 μl) at a rate of 0.05 μl/min. Following infusion, the syringe was left in place for a minimum of 5 min for diffusion of the virus in the tissue. Animals were sutured with dissolvable sutures, and returned to the vivarium where they recovered for three weeks before training in PTC. Approximately six weeks separated the surgical phase from the time PIT testing began. For CeA cannula implants subjects were anesthetized as described above and had stainless steel guide cannula implanted above the CeA (bregma: −2.8 AP, ±4.3 ML, −7.0 DV), which were fixed to the skull using dental cement and jeweler’s screws. Following implantation, subjects recovered for one week before retraining and the second rounds of tests. Perfusions were done within two weeks of the end o*f* testing.

### Perfusions and immunohistochemistry

On completion of the behavioral component of the study, subjects from experiment 2 were perfused for immunohistochemical analysis as described previously (Sears et al., 2013; Ramirez et al., 2015). Briefly, animals were transcardially perfused with room temperature (RT) 4% paraformaldehyde (PFA) in 0.2 M phosphate buffer (PB). Tissue was postfixed 24–72 h in 4% PFA/0.2 M PB and cut into 40 μM sections using a vibrating blade microtome (Leica Biosystems). For assessment of LC infection, CeA cannula placement and axonal DREADD expression in CeA, every 5th section of each structure was processed using a floating immunohistochemistry procedure. Sections were washed with PBS (0.01 M, pH 7.4) at RT three times for 5 min between all steps. Sections were incubated for 30 min in 1% bovine serum albumin (BSA; Sigma) to block nonspecific binding and then incubated overnight (18 h) in primary antibody. Primary and secondary antibody incubations were made in 1% BSA/PBS containing 0.2% Triton X-100.

For LC sections, tissue was incubated in rabbit polyclonal antibody directed at the hemagglutinin (HA) tag fused to hM4Di [1:250 or 1:500; HA-Tag (C29F4) rabbit mAb] and mouse anti-dopamine β-hydroxylase (DBH; 1:2000; clone 4F10.2; EMD Millipore) for 24 h at RT. Tissue was then incubated for 30 min to 1 h at RT in secondary antibodies (1:200; goat anti-mouse Alexa Fluor 488 and 1:200 goat anti-rabbit Alexa Fluor 594; Life Technologies). Sections were mounted on gelatin-subbed slides, and dried briefly (10–20 min) in a dark place. Tissue was briefly rehydrated with a few drops of 0.2 M PB saline and coverslipped with three to four drops of aqueous mounting media (ProLong Gold Antifade Mountant; Life Technologies). Slides were cured overnight at RT and then kept at 4°C before fluorescence imaging and image capture.

For assessment of cannula targeting and axon DREADD expression, sections were incubated in rabbit anti-HA tag [1:500; HA-Tag (C29F4) rabbit mAb, Cell Signaling] at RT overnight. Tissue was then incubated for 30 min to 1 h at RT in secondary antibodies (1:200 goat anti-rabbit Alexa Fluor 594; Life Technologies). Sections were then mounted, coverslipped, and imaged using fluorescent microscopy as described above.

## Results

### β-Adrenergic processes constrain aversive PIT

To determine whether NE contributes to aversive PIT, subjects were treated systemically with the NE β-receptor antagonist propranolol, the agonist procaterol, or the saline vehicle before transfer tests where the effect of the CS on shuttling was measured. These drugs were also tested (via systemic administration) for effects on CS-elicited freezing in a nonavoidance context after PIT testing had concluded. Based on published findings where NE antagonism impaired memory retrieval in the Morris water-maze task (Murchison et al., 2004) we anticipated that propranolol treatment would also impair PIT and that procaterol would enhance the effect.

#### Pavlovian conditioning and USAA training

**Figure 1.**
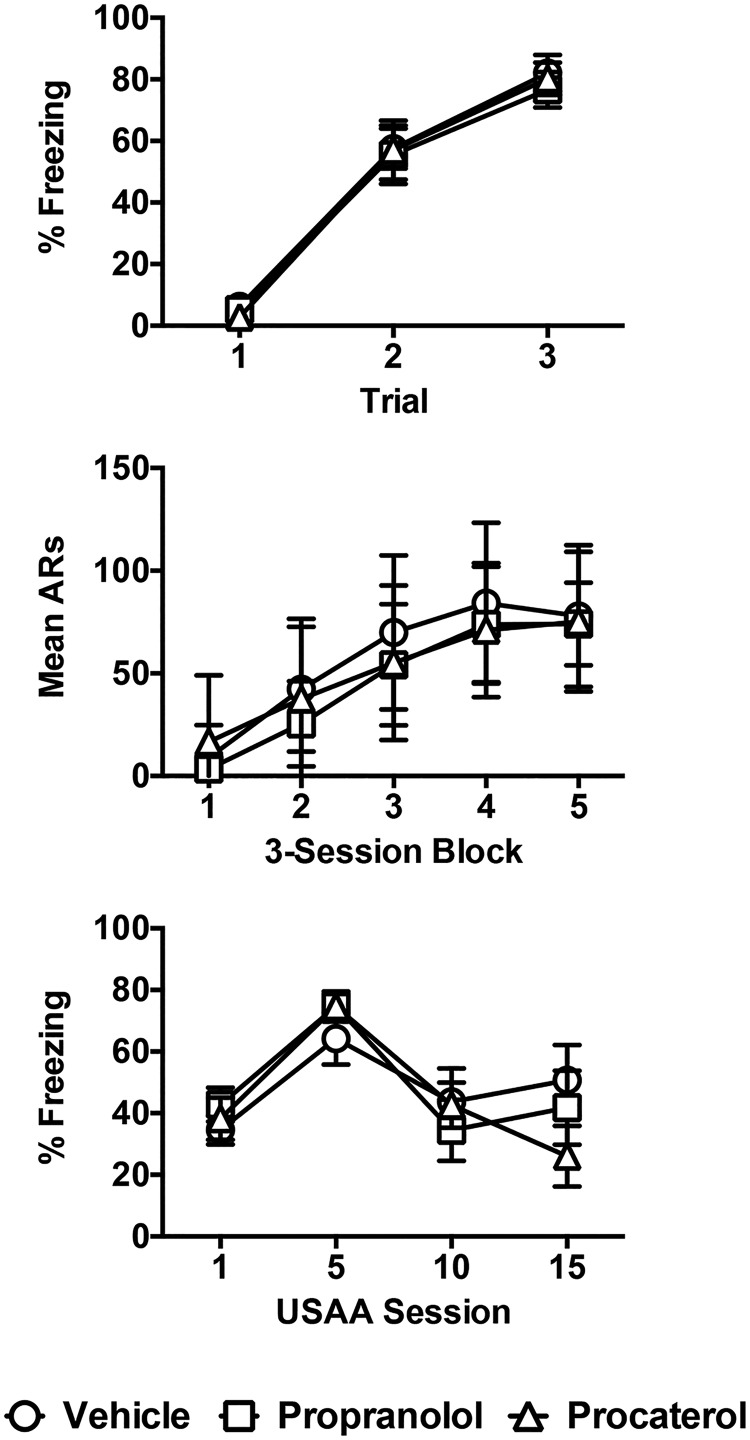
Experiment 1 training data. Percent time freezing to the CS during the PTC phase are presented in the upper panel for each group. Mean avoidance shuttle responses from the USAA phase are presented in the middle panel for three-session blocks of training for each group. Freezing data to the USAA context for the first 5 min of sample sessions (1, 5, 10, and 15) are presented in the lower panel for each group in terms of percent time.

Data from the PTC and USAA phases are presented in Figure 1. A repeated-measures ANOVA on freezing scores during Pavlovian conditioning confirmed that subjects acquired Pavlovian conditioning normally (effect of trial, *F*_(2,94)_ = 444.59, *p* < 0.001). No drug treatments were administered during USAA, and groups were matched based on USAA performance for drug assignment during PIT testing. A split-plot repeated-measures ANOVA on acquisition data from this phase (Fig. 1, middle panel) similarly confirmed that learning proceeded normally during this phase as well (effect of block, *F*_(4,100)_ = 62.03, *p* < 0.001). No significant interaction or main effect was found in this analysis.

Mean percentage time freezing scores to the USAA context during the first 5 min of sessions 1, 5, 10, and 15 of USAA are presented in the lower panel of Figure 1. A repeated-measures ANOVA on these data confirmed that context freezing was comparable for each group entering the test phase (effect of session, *F*_(3,60)_ = 9.63, *p* < 0.01; session × group interaction, *F*_(6,60)_ = 1.45, *p* = 0.21; effect of group, *F*_(2,20)_ = 0.31, *p* = 0.74).

#### Effects of a β-adrenergic receptor (BAR) antagonist and agonist on PIT and CS-elicited freezing

**Figure 2.**
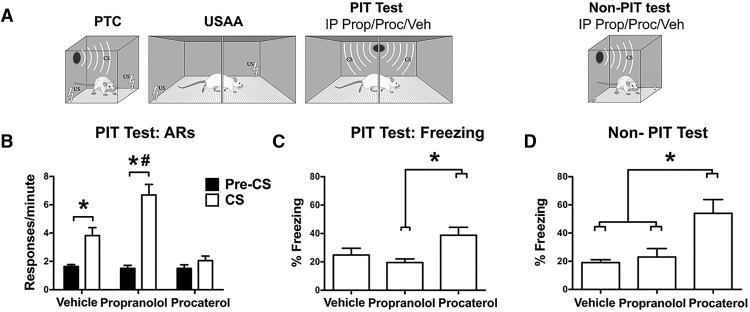
Experiment 1 test data. ***A***, Experimental design, IP stands for intraperitoneal propranolol, procaterol or vehicle treatments administered before tests. ***B***, Shuttling data during the PIT testing phase. These data are presented in terms of responses per minute for each group. Asterisks refer to statistical significance between the pre and CS periods while the hash indicates significance between vehicle and propranolol-treated subjects during the CS (all at the 0.05 α level). ***C***, Freezing during the CS presentations in the PIT testing phase are presented in terms of percent time. The asterisk denotes significant differences in freezing between propranolol and procaterol-treated subjects. ***D***, Freezing during the CS in the follow-up test for CS-elicited freezing conducted in the nonavoidance context. The asterisk signifies statistically significant differences in freezing between procaterol-treated subjects and all other groups.

Shuttling data from the PIT test phase are presented in terms of responses per minute during the pre-CS and CS periods for each group (Fig. 2*A*). Following exclusion of poor performing subjects, final group sizes were eight, nine, and nine for groups treated with vehicle, propranolol, and procaterol, respectively. A split-plot repeated-measures ANOVA on these data found that PIT strength varied among the groups (effect of interval, *F*_(1,23)_ = 54.33, *p* < 0.01; interval × group interaction, *F*_(2,23)_ = 14.71, *p* < 0.01). The main effect of group was also significant, *F*_(2,23)_ = 15.12, *p* < 0.01. Using *post hoc* procedures that use a pooled error term to increase power for detecting interactive effects (Rodger, 1974), PIT (i.e., significant pre-CS vs CS difference) was observed for vehicle (*F*_(1,7)_ = 11.6, *p* < 0.05) and propranolol (*F*_(1,8)_ = 72.5, *p* < 0.05), but not procaterol-treated subjects (*F*_(1,8)_ = 0.86, *p* > 0.05). Direct comparison of vehicle and propranolol CS means revealed a significant effect (*F*_(2,15)_ = 8.34, *p* < 0.05) confirming that while procaterol impaired PIT, propranolol facilitated the transfer effect.

Freezing to the CS during PIT tests can be seen in Figure 2*B* for each group. A one-way ANOVA on these data showed a significant effect of group, *F*_(2,23)_ = 4.97, *p* = 0.017, and Bonferonni corrected *post hoc* tests found that procaterol and propranolol treatments did not significantly influence freezing relative to vehicle subjects (*p*s > 0.05). Although freezing during PIT testing was significantly higher in procaterol than propranolol-treated subjects (*p* < 0.05), both groups showed statistically equal freezing relative to controls. To provide a more accurate measure of conditioned freezing the CS was also tested outside of the avoidance context. As was the case with transfer testing, this was done following systemic drug treatment (Fig. 2*C*). Data were averaged over the three test trials and were analyzed with a one-way ANOVA. Freezing significantly differed among the groups, *F*_(2,23)_ = 8.15, *p* = 0.002, and *post hoc* tests showed that subjects treated with procaterol displayed higher levels of freezing than all other groups (*p*s < 0.05). Vehicle and propranolol-treated subjects showed similar levels of freezing to the CS. In summary, these findings suggest that although crucial for expression of conditioned defensive responses (freezing), NE activity must be inhibited during PIT.

### Activation of LC or LC cell terminals in CeA using designer receptors impairs PIT

**Figure 3.**
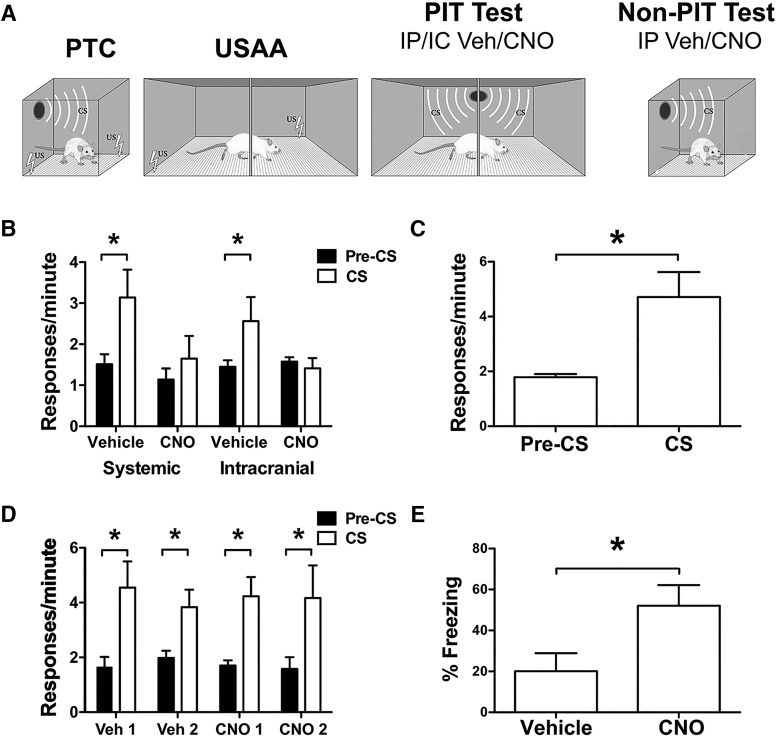
Experiment 2. ***A***, Design for experiment 2 training and transfer tests. ***B***, Data from PIT tests preceded by IP and intracranial CNO/vehicle treatments are presented as a function of drug in terms of responses per minute during the pre and CS intervals. ***C***, Data from control subjects that received IP CNO treatment before PIT testing but were not expressing hM3Dq receptors are also presented in terms of responses per minute. ***D***, Postoperative PIT test data from subjects treated with intracranial vehicle and CNO before sessions without hM3Dq expression. ***E***, The data from the follow-up tests for CS-elicited freezing are presented in terms of percent time freezing and are also expressed as a function of presession drug treatment. Asterisks indicate significance at the 0.05 α level.

Our systemic treatment data suggest that NE plays an inhibitory role in PIT. NE is expressed in the brainstem LC and also plays an important role in aversive processing (Murchison et al., 2004; Gertner and Thomas, 2006; Ferry and Quirarte, 2012). We therefore sought to test the hypothesis that NE from the LC can affect PIT. Gq-coupled hM3Dq DREADDs (Rogan and Roth, 2011) were targeted to NE-expressing LC neurons using the synthetic promoter PRSx8 before behavioral training (Vazey and Aston-Jones, 2014). To activate the designer receptors, CNO was administered before PIT testing, first systemically (IP) and then later, intracranially, into the CeA (Fig. 3*A*). The latter strategy is possible due to the finding that DREADDs are transported to axon terminals, and can be directly activated with intracranial infusions (Mahler et al., 2014; Stachniak et al., 2014; Zhu and Roth, 2014). Excitation of LC via CNO treatment was expected to produce increased NE release and thus give rise to effects on behavior similar to the agonist procaterol in experiment 1, ultimately reducing transfer. Local CNO infusions in CeA were expected to eliminate PIT via projection neurons from LC-CeA. CeA was targeted because it is known to be crucial for expression of both PTC and PIT (LeDoux et al., 2000; Campese et al., 2014).

**Figure 4.**
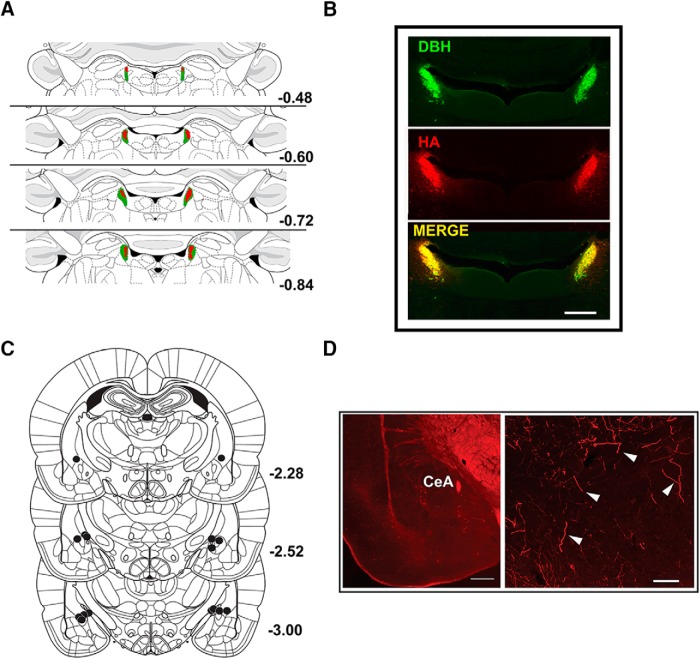
Experiment 2 histology. ***A***, Minimum (red) and maximum (green) viral expression in LC. Values are mm relative to the interaural line. ***B***, Viral expression in LC (HA-tag, red), DBH staining (green), and a merge of the two (yellow; scale bar = 500 μm). ***C***, Infusion sites for intracranial CNO treatment are presented relative to bregma (mm). ***D***, Axon terminal expression of DREADD in CeA (HA-tag, red; scale bars = 500 μm (lower magnification) and 100 μm (higher magnification). HA-immunostaining in CeA is indicated by white arrows.

A repeated-measures ANOVA on USAA data revealed normal acquisition (effect of block, *F*_(4,28)_ = 7.08, *p* < 0.001). Eight subjects met the training criteria and proceeded to the test phase of the study. Data from the PIT testing phase are presented in Figure 3*B* below in terms of responses per minute during the pre-CS and CS intervals. These data were analyzed using a repeated-measures ANOVA which found that systemic and intracranial CNO treatment both impaired PIT relative to vehicle treatment using these routes (effect of interval; pre vs CS: *F*_(1,7)_ = 8.94, *p* < 0.05, drug; vehicle vs CNO: *F*_(1,7)_ = 16.19, *p* < 0.05; interaction, *F*_(1,7)_ = 14.23, *p* < 0.05). The main effect of treatment route (i.e., intracranial vs systemic) was not significant (*F*_(1,7)_ = 0.054, *p* = 0.82), and this factor did not interact with any other factor (drug × route: *F*_(1,7)_ = 0.41, *p* = 0.54; interval × route: *F*_(1,7)_ = 0.72, *p* = 0.43; drug × interval × route: *F*_(1,7)_ = 0.4, *p* = 0.85). Follow-up tests found that the drug × interval interaction was due to significant differences between the pre and CS intervals following vehicle (pre: M = 1.48 95%CI [1.11 1.79]; CS: M = 2.85 95%CI [2.3 3.4]) but not CNO treatment (pre: Mean = 1.36 95% Confidence interval [0.95 1.76]; CS: M = 1.53 95%CI [0.88 2.19]).

In the final phase, tests for CS-elicited freezing were conducted outside of the avoidance context following systemic treatment with vehicle and CNO in a counterbalanced manner. These data are presented in terms of percentage time freezing to the CS following vehicle and CNO treatment for each subject (Fig. 3*E*). Analysis of these data found that systemic treatment with CNO significantly increased freezing compared to vehicle treatment (*t*_(7)_ = 4.28, *p* < 0.01).

To confirm that CNO alone did not impair PIT behavior, eight nonoperated rats received systemic treatment with CNO before PIT testing (Fig. 3*C*). A clear facilitative effect on shuttle responding by the CS was found in these subjects (*t*_(7)_ = 2.90, *p* < 0.05), indicating CNO alone had not affected PIT. Six additional subjects received CeA cannulations following testing for PIT. After recovery these subjects were tested again with CNO and vehicle infusions into CeA using counterbalanced assignments as described above. Data from the postoperative tests are presented in Figure 3*D* and show that CNO infusions into CeA had no effect on PIT relative to vehicle infusions in the absence of viral expression. An interval (pre vs CS) × test (vehicle 1 and 2, CNO 1 and 2) ANOVA found a significant effect of interval (*F*_(1,5)_ = 6.79, *p* = 0.048) with no effect of test (*F*_(3,15)_ = 0.17, *p* = 0.92), and no interaction between the two factors (*F*_(3,15)_ = 0.31, *p* = 0.82).

Together with the systemic drug treatments, these data suggest that NE from LC plays an inhibitory role in CS-elicited active behaviors. Specifically, excitation of LC-CeA connections eliminates PIT and reinstates freezing, nullifying the impact of avoidance learning on CS-elicited behavior. Therefore, to express PIT, NE release in CeA must be blocked.

## Discussion

The underlying psychological nature of avoidance behavior is not very well understood (LeDoux et al., 2016). While this form of learning is sensitive to elements of negative reinforcement such as response feedback and shock omission (Kamin, 1956), other nonreinforcement based accounts have been proposed. For example, avoidance may reflect a special class of unconditioned Pavlovian behavior (Bolles, 1970, 1972) rather than response contingent learning. However, this unresolved issue should not prevent analysis of the effect of the CS on avoidance behavior. PIT is a robust phenomenon that shows a new and unique function of the CS in aversive motivation.

The findings of experiment 1 demonstrate normal facilitation of avoidance by the aversive CS (i.e., PIT) in control subjects. Relative to this group, treatment with systemic propranolol (β-receptor antagonist) enhanced PIT while procaterol (β-receptor agonist) impaired the transfer effect. These findings suggest that NE, acting on BARs, plays an important role in the expression of aversive PIT. While the response feedback cue was presented throughout the test phase this was not likely germane to the effects reported above. Rescorla (1968) has shown that long duration presentations of the feedback cue can suppress avoidance behavior through conditioned inhibitory properties. PIT involves enhanced rates of avoidance responding during the excitatory aversive Pavlovian CS despite continued response contingent feedback cue presentations. Therefore, the effects we report here are likely related to processing the aversive CS and not the feedback stimulus since the modulatory effect on behavior was dependent on the shock-paired tone. It may be that responding at this point in training is not sensitive to feedback (i.e., conditioned inhibition) and is more habitual. Thus, while important for avoidance acquisition, feedback may be of little consequence during transfer. More work would be needed to address this possibility.

Despite this effect on shuttling during PIT, freezing to the CS during PIT for both procaterol- and propranol-treated subjects (while different from one another) was comparable to subjects treated with vehicle. However, in a test better geared to measure CS-elicited freezing without interference from the avoidance response, procaterol treatment significantly increased freezing compared to both vehicle and propranolol-treated subjects. This result suggests that NE transmission to BARs may also be important for the expression of CS-elicited freezing.

Based on findings from Pavlovian conditioning (Bush et al., 2010) and inhibitory avoidance (Ferry et al., 2015) one could expect that freezing might be attenuated by propranolol treatment. Indeed the finding of experiment 1 that procaterol suppresses PIT and promotes freezing CRs supports this idea. If NE release related to CS-processing promotes freezing, then antagonizing BARs should reduce freezing and increase avoidance. The current study did not find reduced freezing in propranolol compared to vehicle subjects, but BAR antagonism did enhance PIT. This is likely because control subjects in this study also showed very little freezing to the CS, as is commonly observed in aversive PIT (Campese et al., 2013). Many published reports find that over the course of avoidance training, freezing CRs are attenuated as avoidance behavior emerges (LeDoux et al., 2016). This is true when avoidance training includes an explicit signal (Cain and LeDoux, 2007; Choi et al., 2010; Moscarello and LeDoux, 2013) as well as in unsignaled avoidance learning paradigms such as that used in the current paper where freezing to the context is reduced over training (Fig. 1*C*; Lazaro-Munoz et al., 2010; Campese et al., 2013). However, because CS-elicited freezing was already low for the control group in the current study after avoidance, any potential impact of propranolol treatment on freezing was obscured by this floor effect. In the absence of USAA learning, propranolol treatment may reduce CS-elicited freezing, though this was not directly evaluated in the studies reported here. Nevertheless, the current findings suggest that changes to NE neuromodulation at BARs during CS processing is an important factor underlying the transition from passive to active defensive responding.

The results of experiment 2 demonstrate that activation of LC using designer receptors impairs aversive PIT. This treatment also increased freezing to the CS in a nonavoidance context. Subjects showed no changes in shuttling response rates during the CS following LC activation via systemic treatment with CNO, but they showed normal facilitation when tested following treatment with vehicle. These findings are in agreement with those from experiment 1, where systemic procaterol was found to eliminate PIT. LC activation with DREADDs produced a behavioral effect similar to procaterol treatment on both shuttling and freezing. This effect was specific to hM3Dq activation and not due to nonspecific effects of CNO (MacLaren et al., 2016). The nonoperated control group treated with CNO before transfer showed intact PIT (Fig. 3*C*). Thus, the effects reported herein are likely due to the activity of designer receptors in NE releasing LC neurons and not CNO alone. While some question has arisen about the nature of ligand-receptor interactions with CNO and DREADDs (Gomez et al., 2017) the findings with control subjects further suggest that peripheral effects of the metabolite clozapine were not responsible for the behavioral effects seen in the study above.

PIT was also eliminated when CNO was infused directly into the CeA, onto the terminals of hM3Dq-expressing LC cells. These same animals showed significant enhancement of responding by the CS when tested following saline infusions. The parameters used for intracranial infusions were based on previous studies that suggest this volume is restricted to CeA. Therefore, it is not likely that CNO spreading to basal amygdala (BA), which also receives LC projections, produced this effect. Furthermore, we have demonstrated that BA is not needed for aversive PIT (Campese et al., 2014). Controls for intracranial treatments were included and showed that CNO infusions into CeA do not impair PIT without viral expression. CeA is required for normal PIT (Campese et al., 2014) and general disruption of processing in this region by infusions or cannulations would be expected to impair transfer. PIT was reduced following surgery (data not included), but not by CNO infusions into CeA relative to vehicle treatment. It should be noted that these subjects were not given retraining after surgery, as LC-viral subjects had been. Thus, the reduction in transfer seen in controls following surgery also likely reflects extinction of PIT over repeated tests. Overall, the results with these controls suggests that the impairment in PIT seen following CNO infusions in LC-viral subjects was due to the effect of CNO on designer receptors (but see Gomez et al., 2017).

This result suggests that that NE transmission between LC and CeA is selectively blocked during PIT. Published findings provide evidence that NE release is involved in aversive memory expression (Murchison et al., 2004; Gertner and Thomas, 2006; Tully and Bolshakov, 2010; Ferry and Quirarte, 2012). Thus, a possible change to CS-processing that arises due to USAA training may be suppression of CS-related NE signaling during retrieval. If CS-elicited freezing requires NE, then this would be necessary to release the animal from response competition and permit the activation of downstream structures that control the shuttle response specifically. The source of this regulation is currently unknown. Some studies have found that regulation of the CeA by prefrontal cortex is involved in acquisition of instrumental avoidance (Moscarello and LeDoux, 2013). PFC is also known to inhibit serotonin-related activity in the dorsal raphe nuclei (Jankowski and Sesack, 2004; Warden et al., 2012), perhaps it has a similar effect on NE release by LC. Therefore, future studies will explore the possibility that this pathway regulates transmission of NE between LC and CeA in some way.

It should be noted that in studies of avoidance learning, poor performance results in the exclusion of subjects. The goal of the studies reported here was to identify key neural components of transition from passive to active behavior when faced with a threat. Because poor performers do not make this transition they cannot be studied to identify these neural circuits. However, they can be used to test hypotheses about the underlying mechanisms. For example, Lazaro-Munoz et al. (2010) found that CeA lesions rescued USAA in poor performing subjects, while Choi et al. (2010) found similar effects in signaled avoidance. These findings suggest that excessive CS-elicited activity in CeA may underlie poor performance. Our current findings suggest that NE signaling in this region may be an important part of this, with poor performers possessing high NE levels and good performers having less transmitted to CeA during recall. This possibility should be further explored by testing the ability of propranolol to rescue avoidance in poor performers. Furthermore, because propranolol increased transfer in experiment 1, this would suggest that the exclusion criteria used here did not result in a sample of subjects with floor levels of NE signaling. If the exclusion criteria had produced this, no significant effects of antagonism should have been observed. This was not the case. While it can be argued that comparable freezing between vehicle and propranolol-treated subjects may be evidence of this, studies of avoidance in general, and PIT specifically suggest otherwise. These studies show that good performing subjects have no baseline deficits in conditioned freezing. Indeed, freezing to the avoidance context during USAA training is normal in experiment 1. What distinguishes poor from good performers is the ability to reduce this freezing and gradually transition to active responding (Lazaro-Munoz et al., 2010).

In summary, these data clearly show that NE is involved in the expression of aversive Pavlovian threat learning, as assessed via PIT. This effect requires inhibition of NE modulation in CeA by projections from LC. This change to CS processing may arise as a direct result of USAA learning to suppress standard freezing CRs to the CS. More work is needed to address this possibility.

## Synthesis

Reviewing Editor: Lorna Role, Stony Brook Decisions are customarily a result of the Reviewing Editor and the peer reviewers coming together and discussing their recommendations until a consensus is reached. When revisions are invited, a fact-based synthesis statement explaining their decision and outlining what is needed to prepare a revision will be listed below. The following reviewer(s) agreed to reveal their identity: Ashleigh Morse.

This manuscript describes evidence of noradrenergic system involvement in cue-motivated avoidance behavior, including localization of its role in the central nucleus of the amygdala. It is believed that this study may provide the first demonstration of a direct LC-CeA circuit mediating aversive PIT, and bi-directional control of the behavior by NE agonism/antagonism. The reviewers were satisfied with the response to prior comments, but there are still issues that remain as critical concerns. After consultation, we have arrived at the following set of issues that must be addressed.

{Please note that the overall ruling is revise, re-review required, rather than reject/ resubmit as we hoped that these issues might be addressable within 2 months time. If the authors feel that these revisions will take more than 2 months to address, please contact the editor and the eNEURO office}

Major Concerns (4):

1. The most significant concern - shared by all of the reviewers -- was the lack of sufficient CNO (DREADD free) controls.

The authors have provided important new control data showing that mere systemic injections of CNO does not reproduce the deficit in PIT performance observed with such treatment in rats expressing hM3Dq in LC-NE neurons. This is important given growing evidence that CNO alone can impact normal behavior and brain function. However, this does not adequately address concerns about the source of the effect of intracranial CNO injections, particularly since such treatment results in much higher CNO concentrations in the region of interest. Given the authors previous work, it seems likely that nonspecific disruption of CeA function could account for this finding.

To address this issue we request the inclusion of additional control experiments to address the effects of intracranial CNO delivery (DREADD- free). Although one can certainly find papers in the literature that lack these essential controls, given the current knowledge, the lack of such additional comparison will be seen as a lack of scientific rigor.

2. More thorough statistical analysis and explication as well as examination of all factors (including interval x drug x injection type)

(a) Currently the authors have only considered the interaction between Interval and Drug, which is the crucial result. The new ANOVA used for Experiment 2 assesses this 2 way interaction. However, the systemic and intracranial injection tests have been pooled together in this analysis. The authors must present evidence that the two routes of administration are indeed equivalent (and hence CAN be pooled). This can be done by conducting a 3-way ANOVA with test/injection type as a factor. If this interaction is nonsignificant, which seems likely, the significant 2-way interval x drug interaction should be unpacked further to identify its source.

(b) The reviews were pleased to see more details about the post-hoc test for Experiment 1 PIT testing, but given that an unconventional approach is being used, further details about the logic behind this approach and how it is implemented should be provided, as has been done by other papers using this strategy. Also, it is unclear why the authors use the strategy outlined by Rodgers 1974 for Experiment 1 but use a more conventional Bonferroni adjustment for the freezing data. Please explain in text as well as in letter of rebuttal.

3. Exclusion of poor performers requires additional discussion and precise justification.

on page 14 it becomes clear that the majority of subjects were excluded due to poor USAA performance - this requires additional attention in the discussion. The authors should consider the possibility that their manipulations may only work for successful "avoiders", which may be rats with some variant of normal NE signalling in the CeA. This may also explain why the authors saw low rates of freezing in the PIT and CS-only tests.

4. The unconventional nature of the PIT test used requires additional analysis, clearer presentation in figures and text and explicit comparison with literature using more conventional procedures.

Of primary importance, the reviewers ask that the authors include additional PIT data to show how shuttling behaviour in the test relates to the first 30s of the CS. Specifically:

a) Given the unusual format of the PIT task, ie. that the CS continues until 10 shuttle responses have been performed, the reviewers request data on when shuttling responses peak after CS onset. Particularly in relation to the first 30s of the CS, as that was its length during training. It may be the case that there is no consistent pattern in any of the drug groups, but shuttling/freezing timing throughout the CS in the PIT test may assist with interpretation of their findings.

b) For clarity, authors should state that there is no feedback given in the PIT test (ie. no flashing light when shuttles have successfully prevented a shock).

c) On page 15 authors state that "direct comparison of vehicle and propranalol means revealed a significant effect" - comparison of which means? CS means? If the CS means were directly compared and found to significantly differ, this should be indicated on the PIT graph in figure 2B for clarity.

d) the freezing means during the PIT test for the propranalol and procaterol groups is significantly different, and should be indicated on Figure 2C.

Additional revisions to text:

In the surgery part of the methods section, the authors don't specify the exact AAV that they used, or the volume infused into the LC. Even if stated in another paper, this information is easy to include in this manuscript and makes this manuscript more useful to those wishing to reproduce their method.
